# Quantitative correlation between the void morphology of niobium-tin wires and their irreversible critical current degradation upon mechanical loading

**DOI:** 10.1038/s41598-018-24966-z

**Published:** 2018-04-26

**Authors:** C. Barth, B. Seeber, A. Rack, C. Calzolaio, Y. Zhai, D. Matera, C. Senatore

**Affiliations:** 1 Department of Quantum Matter Physics (DQMP), University of Geneva, Geneva, Switzerland; 2 Department of Applied Physics (GAP), University of Geneva, Geneva, Switzerland; 3 European Synchrotron Radiation Facility (ESRF), Grenoble, France; 4 Princeton Plasma Physics Laboratory (PPPL), Princeton University, Princeton, NJ USA

## Abstract

Understanding the critical current performance variation of Nb_3_Sn superconducting wires under mechanical loading is a crucial issue for the design of next generation accelerator and fusion magnets. In these applications, the mechanical properties of the conductors may become a limiting factor due to the strong electro-magnetic forces resulting from the combination of large magnets and intense magnetic fields. In particular, the presence of voids in the superconducting filament structure, which are formed during the fabrication and the reaction heat treatment, determines localized stress concentrations and possibly the formation of cracks. In this work, we demonstrate a quantitative correlation between the void morphology and the electro-mechanical limits measured on different Bronze route Nb_3_Sn wires. Hot Isostatic Pressing (HIP) prior to the reaction heat treatment is utilized to partially eliminate the voids. The wires’ void distributions - with and without HIP treatment - are detected and statistically analyzed using high energy X-ray micro tomography. The stress concentration due to the shape and distribution of the voids as well as their impact on the electro-mechanical properties are determined through finite element method modeling. Finally, the results are quantitatively correlated with the experimentally determined limits of the irreversible critical current degradation upon mechanical loading.

## Introduction

The low temperature superconductors (LTS) niobium-titanium (Nb-Ti)^1^ and niobium-tin (Nb_3_Sn)^2^ are the workhorses of superconductivity^3^. They enable nearly every superconducting device^4^, from magnetic resonance imaging (MRI) devices^5^, over fusion^6–8^ and accelerator magnets^9^ to nuclear magnetic resonance (NMR) systems^10^. In particular, Nb_3_Sn, a brittle inter-metallic compound of the A15 phase family^11^ with an upper critical field of about 29 T^12^, is the conductor of choice for generating fields beyond 10 T^13^ [chap. 3.3.2], i.e. beyond the capabilities of Nb-Ti wires. It empowers the high field regions of high resolution NMR spectrometers^10^ and laboratory magnets^14^ as well as some fusion magnets as the toroidal field coils^7^ and the central solenoid^6^ of the International Thermonuclear Experimental Reactor (ITER). With high temperature superconductors (HTS)^15–17^ there is a new class of superconductors that surpass the critical current density of Nb_3_Sn wires in strong magnetic background fields^18^. However due to HTS limited annual production capacities and prices far above any LTS, HTS are not an alternative to Nb_3_Sn wires but a complement outside of the application limits of LTS^19,20^. Nb_3_Sn remains the superconductor of choice for the next large scale science experiments: the future particle accelerators at CERN^21,22^, where unprecedented energies in the region of 100 TeV are envisaged, and the DEMO fusion power plant^23,24^, which is intended to build upon the experience of the ITER. One common feature of these two projects is an enhanced magnetic field intensity combined with an increased current density compared to existing machines. This will result in increased electro-magnetic forces making the Nb_3_Sn wires’ electro-mechanical properties of the highest importance as they can already be a limiting factor in present large scale experiments^25^. In order to build up innovative solutions for improving the electromechanical properties of Nb_3_Sn wires, it is necessary to shed light on the origins and the mechanisms behind their irreversible degradation upon mechanical loading. Beyond the mechanical strength of the constituent materials themselves, two are assumed to be the major factors: the distortion of the A15 lattice resulting from the plastic deformation of the wire matrix^26^ and the presence of voids. In particular, Kirkendall voids formed during the wire reaction degrade the microstructural homogeneity^27^ and cause localized stress concentrations, which act as nucleation points for crack formation^28–30^. The detrimental effect of voids in Nb_3_Sn wires has already been qualitatively observed in investigations of the effect of cyclic mechanical loading on the superconducting properties and the superconducting transition of Cable-in-Conduit Conductors (CICCs) for the ITER project. Reduction of the current sharing temperature^31^ and broadening of the voltage - temperature transition^32^ are observed after high numbers of electro-magnetic force cycles. Cracks leading to the fracture of filament are assumed to contribute to these degradations^33^. Increased crack densities are observed in the proximity of voids through metallographic methods^34^, qualitatively correlating voids with Nb_3_Sn wires’ electro-mechanical limits.

Several methods have been developed at the industrial level to fabricate Nb_3_Sn wires. The most known are the Bronze route^35,36^, the internal tin process^37^ (of which the Restacked-Rod-Process (RRP) is a variant^38^) and the Powder-In-Tube (PIT)^39^ method. Even if the void morphology is influenced by the wire fabrication technology, the constituent materials remain the same. Thus, information about the impact of the voids on the electro-mechanical failure mechanisms can be assumed to be universal for any type of Nb_3_Sn wire. In Bronze route Nb_3_Sn wires, voids are relatively uniformly shaped, homogeneously distributed over the whole wire and they are well isolated. This is in contrast to high-performance wires relying on RRP and PIT manufacturing methods where either large and irregular voids are concentrated to the sub-element areas or huge numbers of tiny voids are contained in clouds confined within the powder core remnants. In order to determine and quantify the impact of the voids on the electro-mechanical properties of Nb_3_Sn wires, one shall be able to change the voids distribution within the superconductor without degrading the current carrying capabilities at zero applied stress. Hot Isostatic Pressing (HIP)^40^ can be employed to this end; prior to, during or after the reaction heat treatment. In particular, the beneficial effects of a heat treatment under hydrostatic gas pressure on the reduction of porosity have been reported by several authors, although critical current after HIP was slightly reduced^41,42^.

In this work, we have been able to use HIP densification to reduce the void fractions of Bronze route wires without degrading their current carrying capabilities. The HIP treatment is performed prior to the reaction heat treatment (“pre-HIP”) at temperatures below the formation of Nb_3_Sn to retain compatibility with the common wind & react device manufacturing methods. Details about this process are reported in the Methods section. In order to correlate the void morphology to the irreversible strain limits of the examined wires, we combine the results of structural and electro-mechanical tests with finite element modeling (FEM). We use high energy X-ray micro tomography to detect and analyze statistically the wires’ void distributions in 3D with and without HIP treatment. In this context, X-ray tomography has distinct advantages over metallographic methods. Firstly, it allows to detect the voids in a large volume of the wire and secondly there is no possibility of creating polishing artifacts which could be mistaken as voids^43^ due to its non-invasive nature. Walters spring measurements provide the limits of the irreversible critical current degradation upon mechanical loading. By using these experimental results as an input for FEM, we find a quantitative correlation between the observed enhancement of the irreversible strain limit and the variation in the void fraction and morphology after HIP treatment. In particular, these results show that the distribution of the mechanical stress inside the wire’s filament bundles is a major factor determining the electro-mechanical properties and open new possibilities for the optimization towards higher mechanical tolerance.

## Results

The examined wires are two types of Bronze route Nb_3_Sn wires, prepared with and without pre-HIP treatment, that present significant differences in void fraction and distribution. Voids in Nb_3_Sn wires can originate from the billet assembly and due to the Kirkendall effect during reaction heat treatment, which occurs when the diffusion rates of chemical species at both sides of an interface are different. During the reaction heat treatment, the diffusion of Sn from Cu-Sn into Nb goes much faster than in the opposite direction (diffusion of Nb into Cu-Sn) resulting in the formation of vacancies that can dissipate or nucleate to voids. In Bronze route wires, the material is subjected to multiple pre-heat-treatments as intermediate annealing steps are required after drawing to counter the work hardening of the Cu-Sn matrix. These anneals could lead to the formation of Kirkendall voids prior to the reaction heat treatment. As the HIP is applied prior to the reaction heat treatment (pre-HIP), it removes voids originating from the billet assembly and the annealing steps. Furthermore it suppresses the formation of Kirkendall voids during the reaction heat treatment by increasing the dissipation chances of vacancies due to the higher wire density.

The first examined wire - type I - is a laboratory made wire of 1.25 mm diameter, developed at the University of Geneva. It consists of 14,641 filaments with an average diameter of 4.5 μm embedded in a high tin content bronze (Cu-15.5Sn) matrix and surrounded by a Nb diffusion barrier. During manufacturing, the wire is annealed at 530–540 °C for 20–25 min each 3 drawing passes. To reach the target diameter the wire undergoes 14 annealing steps; 4 after the first extrusion, 3 after the second extrusion and 7 after the final extrusion. After reaction heat treatment, the void fraction of this wire in the regular - non-HIP - case is as high as 2.1% of the total volume. Wire type II, on the other hand, is a commercial wire made by Bruker EAS for ITER (NSTT8305, ITER ID: 01RBR8305A01C005) in long lengths. It is a 0.82 mm diameter wire with a Ta diffusion barrier. It contains 8,305 filaments of an average diameter of approximately 3.5. The annealing parameters used in the deformation processes are not known. The void fraction in the non-HIP case is only 1.1% of the total volume. The most important characteristics of the investigated samples are given in Table 1.

**Table 1:** Main characteristics of the investigated Nb_3_Sn wire samples.

	Type I: non-HIP	Type I: HIP	Type II: non-HIP	Type II: HIP
wire ID	#43	#43-HIP	#8305	#8305-HIP
manufacturer	UniGe	UniGe	Bruker EAS	Bruker EAS
diameter	1.25 mm	1.25 mm	0.82 mm	0.82 mm
filament count	14,641	14,641	8,305	8,305
filament size	4.5	4.5	3.5	3.5
diffusion barrier	Nb	Nb	Ta	Ta
identifying symbol	red dot	black square	green triangle	blue diamond

### Electro-mechanical properties

**Figure 1:**
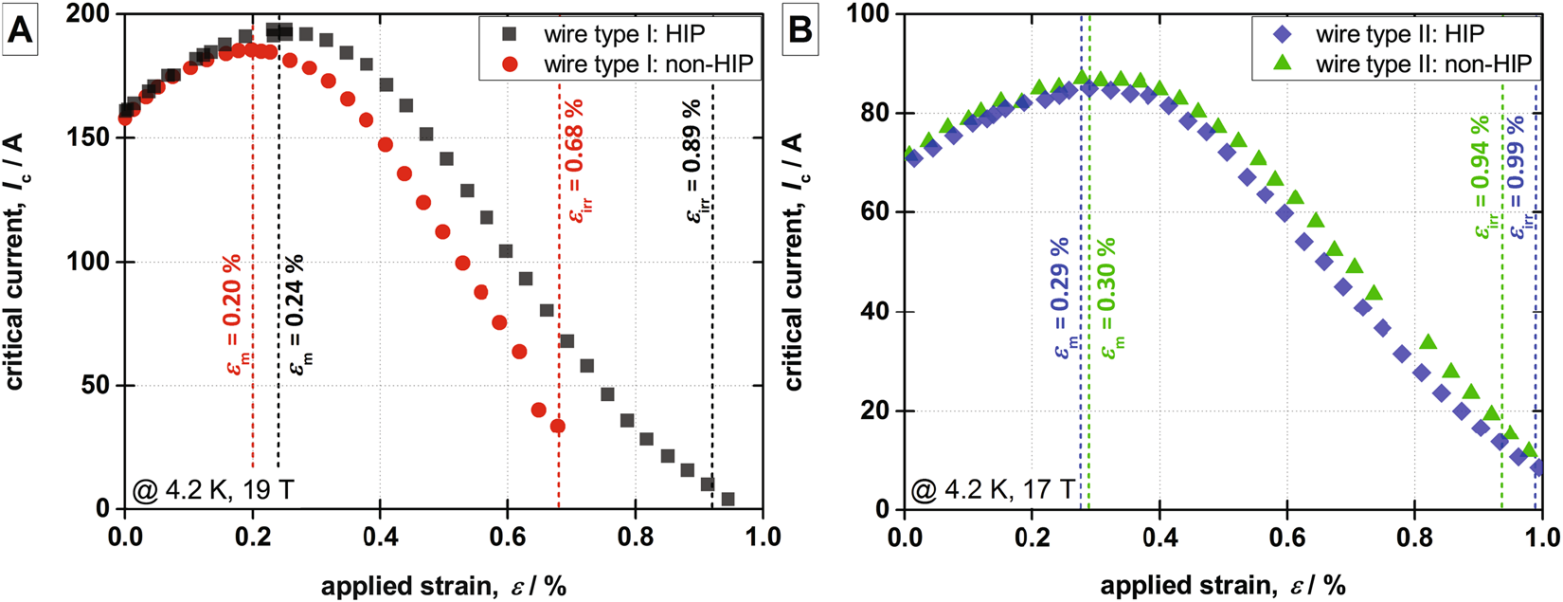
Electro-mechanical properties of the investigated samples as determined with a Walters Spring measurement system: wire type I (**A**) and wire type II (**B**).

Critical current vs. axial tensile strain measurements are performed using a 4-turn Walters Spring (WASP) system, as explained in the “Electro-mechanical measurements” subsection of the Methods section, to determine the irreversibility strain limit of the critical current degradation. Figure 1 reports the critical current versus applied strain curves from wire type I and II, both for the non-HIP and HIP cases. The curves exhibit the well-known bell-shaped appearance, with a maximum corresponding to the value of applied strain *ε*_m_ that releases the thermal precompression of the superconductor so that the Nb_3_Sn unit cell is undistorted^44^. We have defined the irreversible strain limit *ε*_irr_ as the strain level leading to an irreversible degradation of *I*_c_ by 5% upon unloading to *ε*_m_ in the *I*_c_(*ε*) curve. As clarified in the methods section, this criterion was adopted to ease the comparison of the electro-mechanical experiments with the results of the FEM simulations. The HIP treatment influences the electro-mechanical properties differently in the two sample types. This can be best quantified comparing the changes of the critical intrinsic strain *ε*_c_, which is defined as the difference of the irreversible strain *ε*_irr_ and the strain of the maximal critical current *ε*_m_ (see Fig. 1). *ε*_c_ is independent of the pre-compression of the Nb_3_Sn filaments determined by the thermal contraction mismatch in the composite wire upon cool-down. Therefore, it is the ideal parameter describing the samples’ electro-mechanical properties in FEM models, as the cool-down does not have to be taken into account.

In wire type I, the HIP treatment strongly increases the critical intrinsic strain *ε*_c_ from 0.48% to 0.65%; a large enhancement of 0.17%, as shown in Fig. 1A. This is significantly different compared to sample type II, where the HIP treatment only results in a minor improvement of the electro-mechanical properties; *ε*_c_ is solely increased by 0.06% from 0.64% to 0.70%, as shown Fig. 1B. In both sample types, the employed HIP treatment cycle, 200 MPa up to 550 °C for 1 h, as detailed in the “Hot isostatic pressing” subsection of the Methods section, does not affect the current carrying capabilities at zero applied strain. Measurements are performed at 4.2 K in liquid helium bath in a magnetic background field of 19 T for sample type I and at 17 T for sample type II. The lower magnetic field of 17 T is chosen for wire type II as the critical current of this wire goes to zero at 19 T with applied mechanical strains due to the substantially smaller wire diameter of 0.82 mm. Diverging behaviors due to the difference in magnetic background fields can be excluded.

### Void morphology

**Figure 2:**
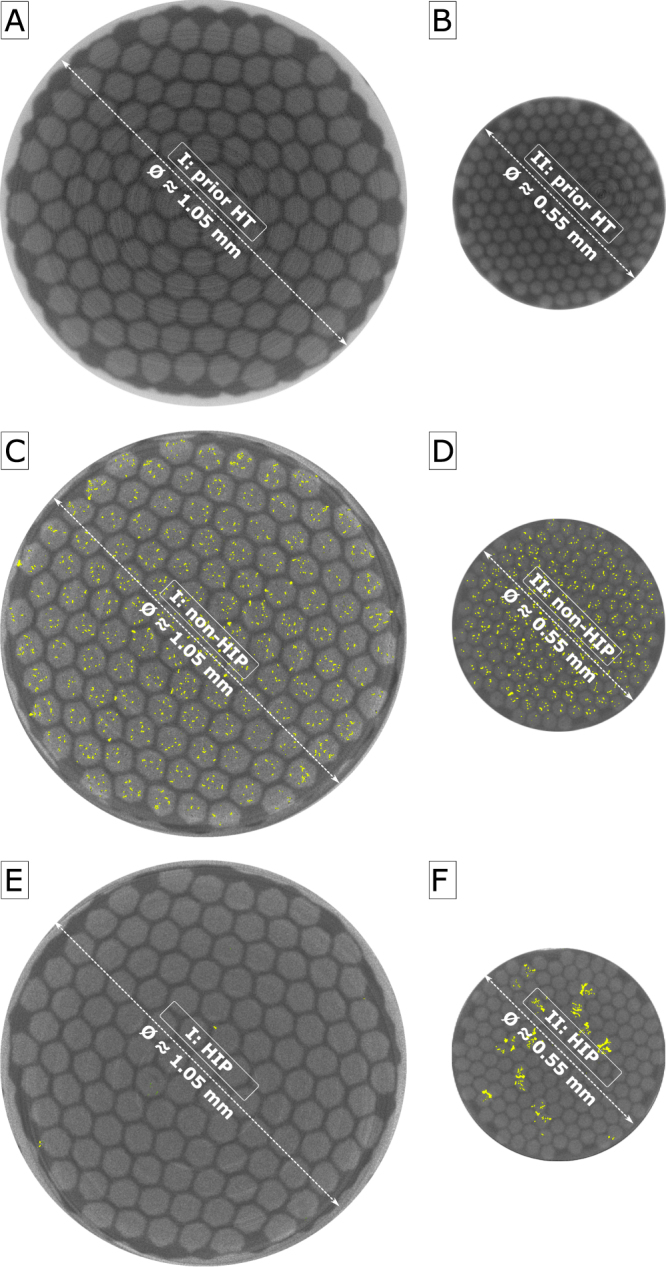
Voids (in yellow) in exemplary slices of the investigated samples without the surrounding Cu stabilizer: wire type I, prior to the reaction heat treatment (**A**), non-HIP (**C**) and HIP (**E**) as well as wire type II, prior to the reaction heat treatment (**B**), non-HIP (**D**) and HIP (**F**).

Synchrotron-based microtomography with high spatial resolution and using hard X-rays (above 80 keV) allows us to detect the voids in 3D in the wire volume with a (sub-)micrometric resolution in an non-invasive, non-destructive way. Further details about the technique are given in the “X-ray tomography” subsection of the Methods section. Both wire types are imaged prior to the reaction heat treatment (prior-HT: A, B), after the reaction heat treatment without hot isostatic pressing (non-HIP: C, D) and after the reaction heat treatment with hot isostatic pressing (HIP: E, F). Prior to the reaction heat treatment, there are no voids visible within the spatial resolution of the experiment (0.6) in both wire types. It is shown exemplary in Fig. 2A,B. This implies that the totality of the investigated voids are formed during the reaction heat treatment either due to the Kirkendall effect or as an agglomeration of trapped gases. Furthermore, this analysis reveals significant differences in the effect of the HIP treatment on the voids in the two sample types. While the void fraction in the non-HIP case of wire type I is high, the value being 2.1% of the total volume (see Fig. 2C), the HIP treatment eliminates the voids almost completely reducing the void fraction by nearly a factor of 40 to 0.05% (see Fig. 2E). On the other hand, the HIP treatment has a much lower effect on the voids in wire type II; it solely reduces the void fraction from 1.1% in the non-HIP case (see Fig. 2D) by a factor of approximately 4 to 0.29% as shown in Fig. 2F. Moreover, the voids in the HIP treated case of wire type II appear as agglomerations of the existing voids, the remaining voids being larger and significantly more elongated. In the non-HIP treated case of both wire types, the voids are homogeneously distributed over the whole cross sectional area meaning that the fraction of voids within the filament bundles, within the matrix and at the interface between filament bundles and matrix correspond to the wire’s geometrical ratios. However, the HIP treatment does not only influences the size and shape of the voids, but it also effects their relative distribution. With HIP treatment, the fraction of voids located at the interface between the filament bundles and the matrix is strongly increased. The probability of a void to be located at the interface is in the HIP treated cases about 3.1 (wire type I) and 5.4 (wire type II) higher than in the corresponding non-HIP treated cases.

**Figure 3:**
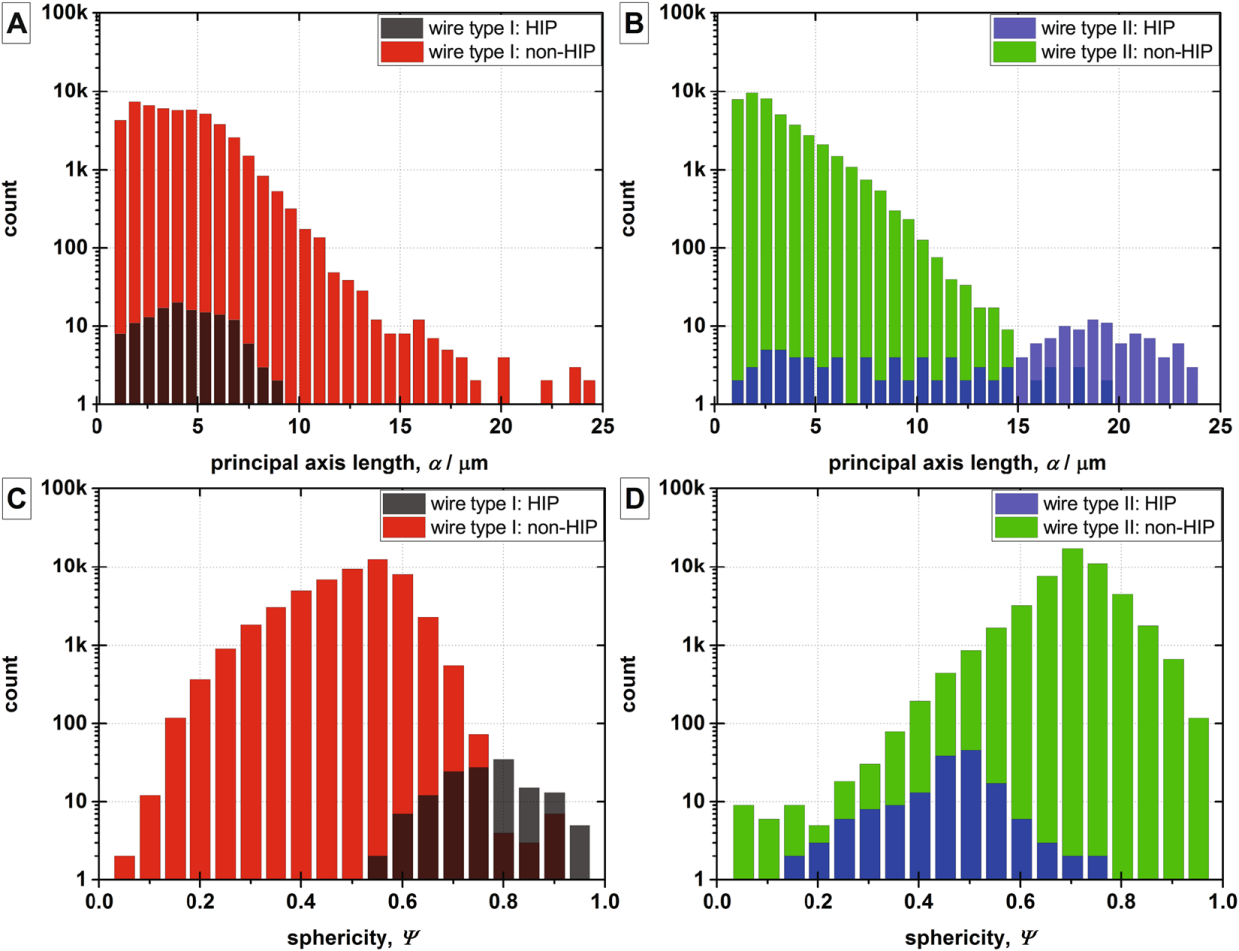
The void morphology of the investigated samples: histograms of the principal axis lengths of the ellipsoid approximated voids of wire type I (**A**) and wire type II (**B**) as well as histograms of the sphericity of the ellipsoid approximated voids of wire type I (**C**) and type II (**D**).

The differences in the impact of the HIP treatment on the wires’ voids are also evident in the four histograms in Fig. 3 that describe statistically the two main geometrical features of the ellipsoid approximated voids: the lengths of the ellipsoids’ major axis and their sphericity. In wire type I, the principal axis lengths *α* (Fig. 3A) are shifted in the HIP case towards smaller lengths, corresponding to smaller void sizes. Furthermore, using the sphericity definition by Wadell (1932)^45^ [p. 736], the average sphericity Ψ_avg_ is increased in the HIP treated case as shown in Fig. 3C. Thus, the few voids remaining in wire type I after the HIP treatment are smaller and more spherical; both aspects reduce their effectiveness as stress concentrators. Combined with a strongly reduced void density, a significant reduction of void related stress concentration can be expected after the HIP treatment for this wire type. On the other hand, in wire type II, the lengths of the principal axis *α* of the ellipsoid approximated voids are shifted towards higher axis lengths in the HIP treated case (Fig. 3B) while the voids’ average sphericity Ψ_avg_ is reduced indicating a stronger elongation (see Fig. 3D). Larger and more elongated makes each of the voids remaining after the HIP treatment of wire type II more effective as a stress concentrator. The reasons behind the diverging effects of the HIP treatment on the void morphology wire type I and in wire type II are not yet fully understood. One explanation could be that this arises from differences in the outgassing of the billets. The billets of the laboratory made wire (wire type I) were outgassed at 200 °C under 10^−5^ mbar vacuum for 24 h before sealing by electron-beam welding. It is likely that the outgassing of the industrial wire (wire type II) was carried out for shorter time or under a less good vacuum, resulting in a higher residual gas content. At the end of the HIP treatment or during the reaction heat treatment of the wire, the trapped gas may nucleate again into larger voids while the wire is at high temperature and low pressure. Because of this, the HIP treatment can be less effective in wires with a high amount of trapped gas. The different void morphologies of the four samples match qualitatively with their electro-mechanical properties. The large enhancement of the critical intrinsic strain *ε*_c_ after the HIP treatment of wire type I corresponds well with a significant reduction of the void fraction as well as a change towards a shape of the voids with low stress concentration effectiveness. Wire type II’s low increase of *ε*_c_ after the HIP treatment is in line with a lower reduction of the void fraction and a shape of the remaining voids with high effectiveness as stress concentrator.

The drop in count towards the lowest principal axis length bin size in the non-HIP cases of both wires (see Fig. 3A, red histogram and Fig. 3B, green histogram) is an effect of the digital filtering of the voids detected in the tomography data. From an extrapolation of the histogram towards the lowest principal axis length, the filtering is estimated to result in about 6% total count reduction. As this corresponds to less than 1% of the total void volume, it has negligible effect on the obtained size and shape distributions as well as on the FEM models based on these statistical data.

### Correlation between the void morphology and the electro-mechanical properties

From the post-processing of the micro tomography data (see Methods, “Void detection and analysis” subsection), we extract a statistical description of the voids in terms of shape and distribution: the axis lengths in the ellipsoid approximation, its orientation with respect to the wire axis as well as the distance of its “center of mass” to the nearest neighbors. Furthermore, location distributions are calculated relative to the wires’ internal features by comparing the wires’ geometries with the fractions of voids located within the filament bundles, within the matrix and at the interface. All parameters are collected into histograms and they are used as input for the void generation in the FEM models to mirror the void morphology of the samples as closely as possible. As detailed in the methods section “Mechanical finite element method model”, there are two placement algorithms for voids: algorithm “A” for homogeneously distributed voids and algorithm “B” for inhomogeneous void distributions.

Thin disks of the wires are modeled with FEM; the voids are added as ellipsoids in an iterative algorithm respecting the statistic sizes, orientations and distances to the nearest neighbors from the X-ray tomography analysis of the corresponding wire till the target void fraction is reached. This FEM simulation, all material and mesh properties as well as the critical intrinsic strain determination method are reported in detail in the “Mechanical finite element method model” subsection of the Methods section. Identical criteria are employed to determine the critical intrinsic strains in the measurements and in the FEM models.

**Figure 4:**
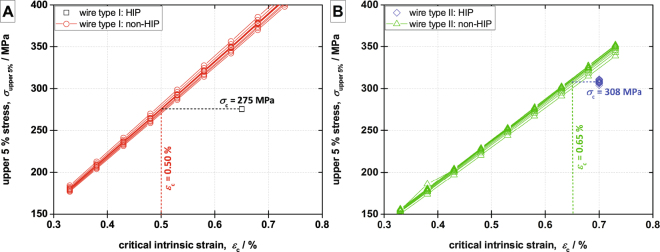
Change of the critical intrinsic strain due to the change in the void distribution and morphology, from HIP to non-HIP, in wire type I (**A**) and in wire type II (**B**).

For both wire types, their HIP treated case is used as the baseline. The void distributions are inhomogeneous, therefore, placement algorithm “B” is used taking the relative locations of the voids into account. It respects the fractions of voids located within the filament bundles, within the matrix or at the interface as determined from the X-ray tomography. In the FEM simulations, we apply to the wires their critical intrinsic strains *ε*_c_, as determined from the electro-mechanical measurement, through a displacement boundary condition to determine the critical stress value. In the case of HIP treated samples, the values of *ε*_c_ are 0.65% and 0.70% for wire type I and II, respectively. The baselines are modeled 12 times, each run with a unique distribution of the voids, and the von Mises stress distribution within the Nb_3_Sn bundles is determined. 12 separate runs of the model are deemed sufficient to cope with the variations of the statistical void distribution in order to gain sound averaged values. Due to the very low void density in the HIP treated case of wire type I, the differences between the different runs of the model are negligible. Even a separate run of the model without any voids yields the same von Mises stress histogram. For each sample we calculate the cumulative von Mises stress distribution in the Nb_3_Sn filament bundles. For a given stress value, the distribution provides the percentage of Nb_3_Sn area under a stress value lower or equal. Assuming that a certain percentage of irreversible degradation of the critical current corresponds to the same amount of Nb_3_Sn cross sectional area reduction, we adopt the 5% degradation of the current upon unload as critical limit. The corresponding value of the upper 5% stress, *σ*_upper5%_, obtained from the cumulative volume-weighted von Mises stress map, is 275 MPa in all runs of the model for the HIP-treated wire type I. Thus, we consider 275 MPa as the critical stress of the filaments in wire type I. This stress value is an intrinsic property of the Nb_3_Sn filaments in wire type I and it remains identical in the HIP and the non-HIP cases. In the following, the non-HIP case of wire type I is simulated for applied strains from 0.33% to 0.73% using a 0.05% step size. Due to a homogenous void distributions in the non-HIP treated cases of both wire types, void generation algorithm “A” is used. This algorithm places the seeds randomly over the whole modeled volume and does not consider the locations of the voids relative to the wire’s internal structure. As before, the void generation, the model and the post-processing are run 12 times. However, in the non-HIP case, distinct differences in the von Mises stress histograms and in the *σ*_upper5%_ are obtained due to the randomness of the void distributions (see Fig. 4A). At the same applied strain, the *σ*_upper5%_ values are increased compared to the HIP case, clearly highlighting the effect of the stronger stress concentration due to the significantly higher void fraction and unfavorable shapes of the void in the non-HIP case. Searching for the applied strain of the non-HIP model with a *σ*_upper5%_ of 275 MPa yields the model’s prediction of the critical intrinsic strain in the non-HIP case. As shown in Fig. 4A, we find over the 12 runs of the non-HIP model of wire type I a critical intrinsic strain range of 0.490% to 0.511% with an average of 0.50%. Thus, the model predicts a reduction of the critical intrinsic strain by 0.15% as a consequence of the voids acting as stress concentrators, which is in a good agreement with the 0.17% difference obtained in the electro-mechanical WASP measurements (see Fig. 1A) for this wire type.

Wire type II is processed identically. The HIP treated case with *ε*_c_ = 0.70% is used as a baseline. Over 12 runs of the model for the the HIP-treated wire, a *σ*_upper5%_ of 308 MPa is obtained with a uncertainty of 2.1. Compared to wire type I, the higher remaining void fraction in the HIP case of wire type II results in stronger deviation between the runs of the model. 12 runs of the model for wire type II in the non-HIP case with applied strains in the interval from 0.33% to 0.73% predict critical intrinsic strains in the interval from 0.640% to 0.666% with an average of 0.65%, as shown in Fig. 4B. This results in a reduction of the critical intrinsic strain by 0.05% from the HIP to the non-HIP cases that is again in good agreement with the 0.06% difference observed in the electro-mechanical WASP measurements for this wire type.

To summarize, in both sample types, a change in the void morphology can be correlated quantitatively to the experimentally observed variation in the electro-mechanical properties, as a consequence of the stress concentration at the voids.

## Discussion

A correlation between the voids and the electro-mechanical properties can already be seen qualitatively comparing the results of the electro-mechanical tests with the X-ray tomography images. It is validated by the histograms that describe statistically the void morphology making clear that not only the total void fraction in the wire but also the shapes of voids play a major role. FEM models, using the void morphology obtained from the X-ray tomography as input, predict a reduction of the irreversible strain limits due to the voids by 0.15% for wire type I and by 0.05% for wire type II. These are in very good agreement with the electro-mechanical WASP measurements that yield reductions by 0.17% and by 0.06%, respectively. Even though this quantitative analysis method solely relies on stress concentration at the voids, it can still reproduce closely the experimentally observed behaviors. The relative deviations between measured and predicted values are for both wire types less than 5%. The stress concentration at the voids can therefore be assumed to be the at the origins of the irreversible critical current degradation upon mechanical loading in Bronze route Nb_3_Sn wires. This investigation is focused on single wires exposed to solely longitudinal tensile strains. In such cases, the strain state of the whole wire is well defined allowing a precise determination of localized strain changes due to stress concentration at the voids. As the FEM model’s predictions closely match the experimentally determined values of *ε*_irr_ it is evident that other mechanisms such as local reductions of the thermal pre-compression around the voids play only secondary roles. In large scale application however, the wires are in general assembled into cables adding additional layers of complexity. In such cables, the wires are subjected to different mechanical strains and stresses, which can even vary periodically along the length of the wire. In the Rutherford cables of accelerator magnets and in CICCs of fusion magnets, pre-compression, thermal stresses, bending and transverse stresses affect the conductors in addition to the here investigated longitudinal strains. This renders the wire’s strain state inhomogeneous even without considering any voids. Because of this, predictions for the performance of superconductor cables are challenging, as a large number of different effects have to be considered. Nevertheless, the here presented densification and modeling techniques clearly show that a reduction voids in Nb_3_Sn wires results in an enhancement of the electro-mechanical properties. This result is not self evident as there are well documented cases on other superconductor materials were the void morphology and the electro-mechanical properties are clearly uncorrelated. E.g. bismuth-strontium-calcium-copper-oxide 2212 (Bi_2_Sr_2_CaCu_2_O_8+x_ or Bi2212) round wires^17,46^ exhibit an enormous void fraction if reacted at ambient pressure. These voids can be almost completely suppressed leading to a nearly total densification of the wire^47^ if the heat treatment pressure is increased to 100 bar^48,49^. The change in voids is even larger than what is achieved on Bronze route Nb3Sn wires. One would assume that the increased heat treatment pressure strongly enhances Bi2212 wires’ electro-mechanical properties. However, different investigations in longitudinal tension, in longitudinal compression and in transverse compression show only minimal, if any benefits^47,50,51^. In Bi2212 round wires, the voids are clearly not the dominating factor limiting the electro-mechanical properties. Thus, in technical superconductor wires, the effect of the voids on the degradation of the critical current upon mechanical loading cannot be taken for granted. In contrast to the behaviour of Bi2212 round wires, we have shown in this work a quantitative correlation between a change of the void morphology with a shift in the electro-mechanical properties in Bronze route Nb_3_Sn wires. We found that the stress concentration at the voids is the dominant mechanism for the irreversible critical current degradation. This shows the importance of the microstructure for the optimization of Nb_3_Sn wires towards higher mechanical strain highlighting that the distribution of the mechanical stresses inside the wire’s filament bundles is a major factor determining the electro-mechanical properties. In this work, we have shown that hot isostatic pressing prior to the reaction heat treatment (pre-HIP) can enhance the electro-mechanical properties without negatively influencing the current carrying capabilities at zero applied strain. Pre-HIP treatments, at temperatures below the formation of Nb_3_Sn, retain full compatibility with common “wind & react” or “wind, react & transfer” device manufacturing methods^4^, they are feasible in large scale applications and are therefore one possibility for wire optimization towards higher mechanical strength. Furthermore, with stress concentration being the dominant mechanism, one can assume that layouts which diverge the mechanical stress from the filament bundles or distribute the stress homogeneously over the whole wire to avoid stress peaks should also boost the electro-mechanical performance. In any case, information about the origin and the mechanism behind the irreversible degradation upon mechanical loading may enable manufacturers to tune their processes accordingly.

**Figure 5:**
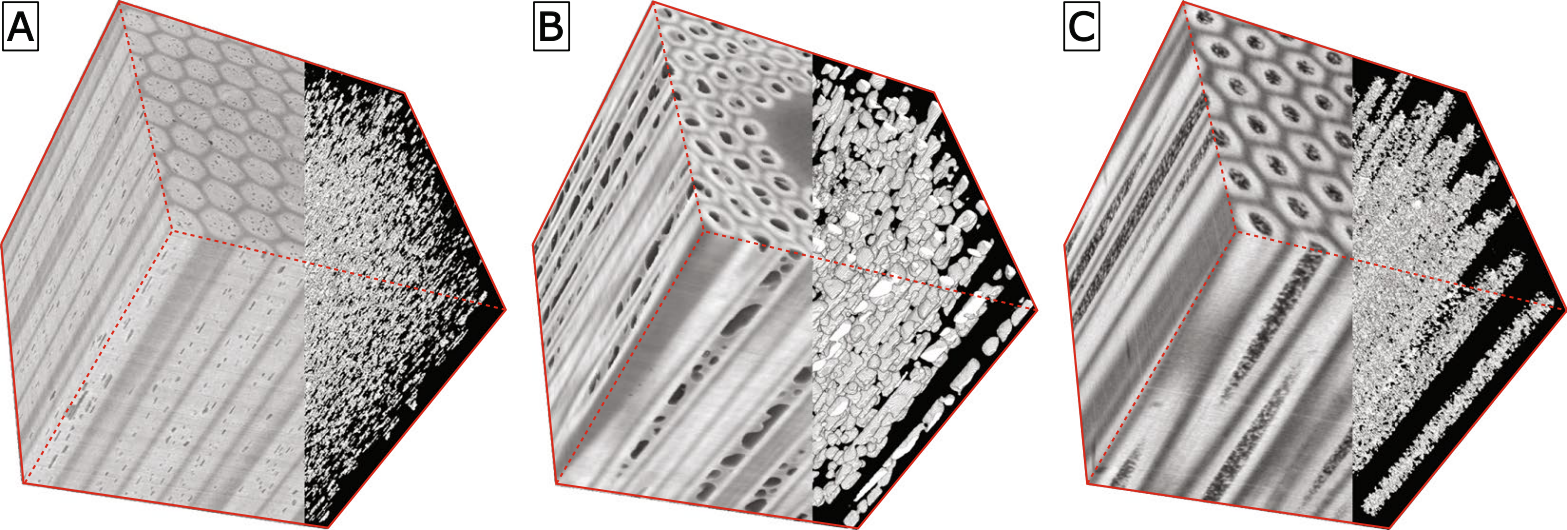
Exemplary 3D assembled X-ray tomography images in a cube of Bronze (**A**) RRP (**B**) and PIT Nb_3_Sn wires (**C**).

While a clear quantitative correlation of the void morphology and the electro-mechanical properties appears in wires manufactured by the Bronze route, it remains a question for the other types of Nb_3_Sn wires: internal tin or restacked-rod-process (RRP) and powder-in-tube (PIT) wires. Their void morphologies are fundamentally different compared to Bronze route wires, as visible in 3D assembled X-ray tomography images in Fig. 5, making a different approach necessary. While in Bronze route wires small, regularly shaped and well isolated voids are distributed over the whole wire, the voids in RRP wires are very large and irregularly shaped; they are restricted to the wire’s sub-elements. In PIT wires on the other hand, there are huge numbers of very small voids which are clustered in large clouds within the volume contained in the tubes. These differences exclude a direct transfer of the results of this study to RRP and PIT Nb_3_Sn wires. As the constituent materials remain the same, the voids can still be assumed to play a major role in the electro-mechanical properties of RRP and PIT Nb_3_Sn wires. Due to generally lower irreversible strain limits in these wires compared to Nb3S wires manufactured with the Bronze route^52^, any improvement of the electro-mechanical properties is even more important. Void reduction and densification with HIP treatment could be a valuable tool for the manufacturers of such wires. In addition, the modeling method proposed in this work can still be used to predict the possible enhancement of the irreversible strain limits in RRP and PIT wires following a reduction of the void fraction.

## Methods

### Hot Isostatic Pressing

Hot isostatic pressing (HIP) is a manufacturing process used to reduce the porosity of metals and to increase the density of many ceramics with the goal of enhancing the mechanical properties and the workability. In a high pressure containment vessel, a material is subjected to both elevated temperature and a high isostatic gas pressure of an inert gas, mostly argon (Ar) to prevent chemical reactions. In general, the containment vessel is equipped with heating elements and temperature sensors while the gas pressure is generated through a combination of pumping with a compressor and gas expansion with increasing temperature^40,53^. There have been several efforts to utilize HIP treatments in the densification of superconductors in different configurations: HIP treatments during, after (“post-HIP”) and before (“pre-HIP”) the reaction heat treatment have been investigated with various degrees of success^41,42,54–56^. In general, HIP treatments during the reaction heat treatment results in general in diminished current carrying capabilities, while post-HIP treatments are unpractical for large magnet systems and reduces the compatibility with the “wind & react” or “wind, react & transfer” device manufacturing methods^4^. The HIP treatment therefore has to take place before the reaction heat treatment; however, the temperature has to be kept low enough to prevent the formation of Nb_3_Sn.

In this work, an ASEA HIP machine (today Quintus Technologies), model QIH-9 with a Kanthal furnace is employed^57^. It allows temperatures of up to 1200 °C and pressures of up to 200 MPa. We successfully densify Bronze route Nb_3_Sn wires without degrading their current carrying capabilities using the following pre-HIP cycle. In a first step, pressure is built up to about 100 MPa by pumping with the compressor at room temperature. Then heating is switched on with a rate of 50 °Ch^−1^ which is common for Nb_3_Sn conductors. During ramping up the temperature the pressure is first linearly increased and then kept constant upon reaching 200 MPa. Once the target temperature of 550 °C is achieved, the hold time is 1 hour. The temperature can be kept constant by 0.5 °C. Then the pressure is released and the system is cooled down to 150 °C, using again a temperature ramp rate of 50 °Ch^−1^, followed by switching-off of the furnace.

### Electro-mechanical measurements

The electro-mechanical properties of all samples are determined using a 4-turn Walters Spring (WASP) system with a total measurement length of 486 mm. The superconductor wire sample is soldered on the outside of a 39 mm diameter titanium-aluminum-vanadium (Ti-6Al-4V) spring^58^. Loading and unloading of the spring changes its diameter straining the sample axially in tension or in compression.

The strain is applied in identically spaced steps with a critical current *I*_c_ measurement at each step. The strain of the maximal critical current is obtained by fitting the average critical current vs. strain behaviour of the 4-turns of the Walters spring with a fifth order polynomial expression and calculating the maximum. In order to determine the samples’ irreversible strain limit, *ε*_irr_, the strain *ε*_n_ is partially released to the strain of the maximal critical current *ε*_m_ after each critical current measurement *I*_*c*_(*ε*_*n*_) in the tensile region. At *ε*_m_, the critical current ${I}_{c}^{{\rm{back}}}({\varepsilon }_{{\rm{m}}})$ is measured again; this is referred to as the “backsteps”. In the reversible strain region of the wire the critical current of these backsteps is increasing with increasing strains *ε*_n_ due to the plastic deformation of the matrix material, leading to hydrostatic stress release and favorable increase of Nb_3_Sn’s lattice parameter and unit cell size^44,59^. From a certain strain *ε*_n_*, the critical current of the backsteps is decreasing, due to damage to the superconductor filaments. Such a reduction can be used as the criteria to determine the irreversible strain limit^59^ of the wire. In this work however, we employ a modified criteria to ease the comparison with the numerical FEM simulations. Therefore we have defined the irreversible strain limit *ε*_irr_ as the strain from which the backstep results in a 5% lower critical current than the absolute maximal critical current of all the backsteps. This is in line with the determination of the irreversible strain the FEM simulations, where *ε*_irr_ is defined as the strain at which 5% of the superconductor cross sectional area is exceeding the stress limits of the material. The reduction by 5% has been chosen for two reasons: firstly it corresponds to the commonly used 5% critical current degradation limits^26,47^ in other types of electro-mechanical measurement and secondly it has proven a suitable threshold value for numerical modeling where lower values can strongly increase the variance due to numerical noise. In all presented electro-mechanical measurements, the pre-strains due to thermal expansion mismatch of the sample and spring are determined and subtracted^58,60^.

### X-ray tomography

X-ray micro tomography is a non-invasive, non-destructive method allowing the determination and the discrimination of internal features without influencing the sample in in a widely non-destructive manner. During the acquisition, the sample is fixed on a rotating holder in a parallel or divergent X-ray beam. A detector behind the sample registers its X-ray absorption which is correlated to the weight of the containing elements and the density. The sample is rotated 180 or 360 while measuring the X-ray absorption in small angle intervals. Each of these projections corresponds to the sample’s absorption in a certain spatial direction; together, these projections can be assembled into a three dimensional (3D) X-ray absorption map of the sample. Dark-field correction and flat-field correction are performed by subtraction of the background signal, correction of the nonlinearities of the detector and the lenses as well as normalization of the measured intensities. Polychromatic, hard X-ray synchrotron radiation as proven to be excellently suited to study the internal features of dense materials such as superconductors with high spatial resolution^61^.

In this work, X-ray tomography is performed with a photon energy of 89 keV using a 2560 × 2120 pixel resolution detector at beamline ID19 of the European Synchrotron Radiation Facility (ESRF) in Grenoble, France with a experimental setup similar to^61^. For each acquisition, the sample is rotated 360 °C registering a projection each 0.012° (30000 projections in total). A spatial sampling of 0.57 μm/pixel is applied.

### Void detection and analysis

In the first step of the post-processing of the X-ray tomography data, the void areas inside the tomography volume are detected from a tomography data set. The detection is done slice by slice using the commercial software package MatLab; each of the slices is cropped and masked to restrict the following void detection and void enhancement steps to the areas containing the sample. Each pixel in the masked area is compared with a threshold which is adjusted to the slice’s average brightness and the mean brightness in the region surrounding the pixel to determine if the pixel is a void. This adaptive threshold^62^ yields significantly higher detection accuracy than fixed thresholds as variations of the brightness of the images are taken into account. The results of these comparisons form a binary 3D matrix which is in the following referred to as the “void matrix”. Each entry in the void matrix corresponds to a volume element in the investigated sample of a border length of 0.57 μm with a “1” signifying void and “0” non-void. Afterward, a second adaptive threshold is used to distinguish between the filament bundles and the wires’ matrix. A three-dimensional binary map of the matrix is assembled. The position of each detected void is correlated with this map to determine if the void is located within a filament bundle, at the interface or within the matrix. A volume threshold of 20% is imposed. This means that if less than 20% of a void volume coincides with the map of the matrix, the void is attributed to the filament bundles. A coincidences of more than 80% makes it a matrix void. In any other case, the void is considered an interfacial void. The obtained fractions of filament bundle, matrix and interfacial voids are compared with the wires’ geometrical fractions to determine the void location distribution relative to the internal features.

In the second step of the post-processing, the detected void pixels are digitally filtered and grouped into connected void areas which are processed with repeated open and close morphological operations based on Euclidean distance maps^63^. To remove “dark noise”, i.e. isolated pixels in a void region which have not been detected as voids, a distance transformation^64^ with a threshold of a few pixels is applied to the void matrix. For all void pixels, it sets all adjacent pixels within the 3D distance of the threshold or less to “1”. It grows all void regions in the void matrix by the distance threshold. Afterward, applying the distance transformation with the same threshold on the negated void matrix shrinks the void regions by the distance threshold back to their original size. The dark noise however is now eliminated. To remove “bright noise”, isolated void pixels outside of void regions, all void pixels are grouped into connected void areas^65^. The number of elements^66^ [parameter “Area”] of each connected void area is compared with a size threshold and tiny void areas, the bright noise, are discarded.

In the third step of the post-processing the shape, the size and the relative position of the connected void areas are analyzed by an approximation with ellipsoids. Such an approximation strongly increases the regularity by reducing the shape complexity of the voids, a necessary step in order to obtain void data adequate for FEM model input. Ellipsoids are determined to be the ideal simple geometric shape for Bronze route wires. Using the procedure described in detail in^67^, the dimensions of an equivalent ellipsoid are determined. Histograms of the ellipsoids’ main parameters, the distances to the nearest neighbors and the void volume fraction are the input for the following mechanical FEM models.

### Mechanical finite element method model

To determine the effect of the voids as stress concentrators, a 0.1 mm thick disk of the wire is modeled with the finite element method in real dimensions using the solid mechanics component of the commercial software package Comsol Multiphysics^68^. Internal features of the wire are implemented down to the filament bundles, a resolution similar to what is achieved which the X-ray tomography. The models consider mechanical stress as a trigger for cracks, but neither the propagation nor the stopping of these cracks. These additional features would require more detailed models (down to the filaments)^69^ and rely either on dynamic re-meshing^70^ or on the extended finite element method (X-FEM)^71^ to cope with geometric discontinuities and the asymptotic conditions near the crack tip. In terms of computational power requirements and model convergence this is not feasible in 3D for very high numbers of voids (the models of wire type I, non-HIP contain of the order of 2000 voids).

The voids are added to the model as ellipsoids in iterative algorithms with distances, sizes, shapes and orientation taken from the histograms of the X-ray tomography analysis. Two void generation algorithms are implemented: algorithm “A” for homogeneous void distribution and algorithm “B” for inhomogeneous void distribution. Both algorithms place the first 10% of the voids randomly as seeds, preventing that a void is generated in a volume which is already occupied by another void. The remaining voids are placed iteratively at a distance to already existing ones that is statistically obtained from the X-ray tomography analysis. The iterative placement stops as soon as the overall void fraction of the modeled wire type is reached. Only placement algorithm “B” (for inhomogeneous void distributions) is aware of the internal geometry of the wire. It respects the fractions of voids within the filament bundles, within the matrix and at the filament bundle - matrix interface as determined from the X-ray tomography. Nb_3_Sn and Nb are considered in the elastic regime while Cu and Cu-Sn are described elasto-plastically using bi-linear isotropic material properties to account for their early mechanical yielding at cryogenic temperatures. The void regions are modeled as empty volumes. All material properties used in the model are taken from^72–74^ and are summarized in Table 2.

**Table 2:** Summary of the material properties used in the mechanical FEM models.

	Cu	Cu-Sn	Nb	Nb_3_Sn	Ta
Young’s modulus	110 GPa	100 GPa	105 GPa	90 GPa	185 GPa
yield stress	1 MPa	344 MPa	—	—	—
tangent modulus*	1.300 GPa	1.084 GPa	—	—	—
behavior	elasto-plastic	elasto-plastic	elastic	elastic	elastic
Poisson’s ratio	0.35	0.34	0.4	0.36	0.34
density	8960 kg m^−3^	8200 kg m^−3^	8570 kg m^−3^	8300 kg m^−3^	16400 kg m^−3^

*In the bi-linear isotropic material description, the tangent modulus is the slope of the stress-strain curve after the yield stress.

**Figure 6:**
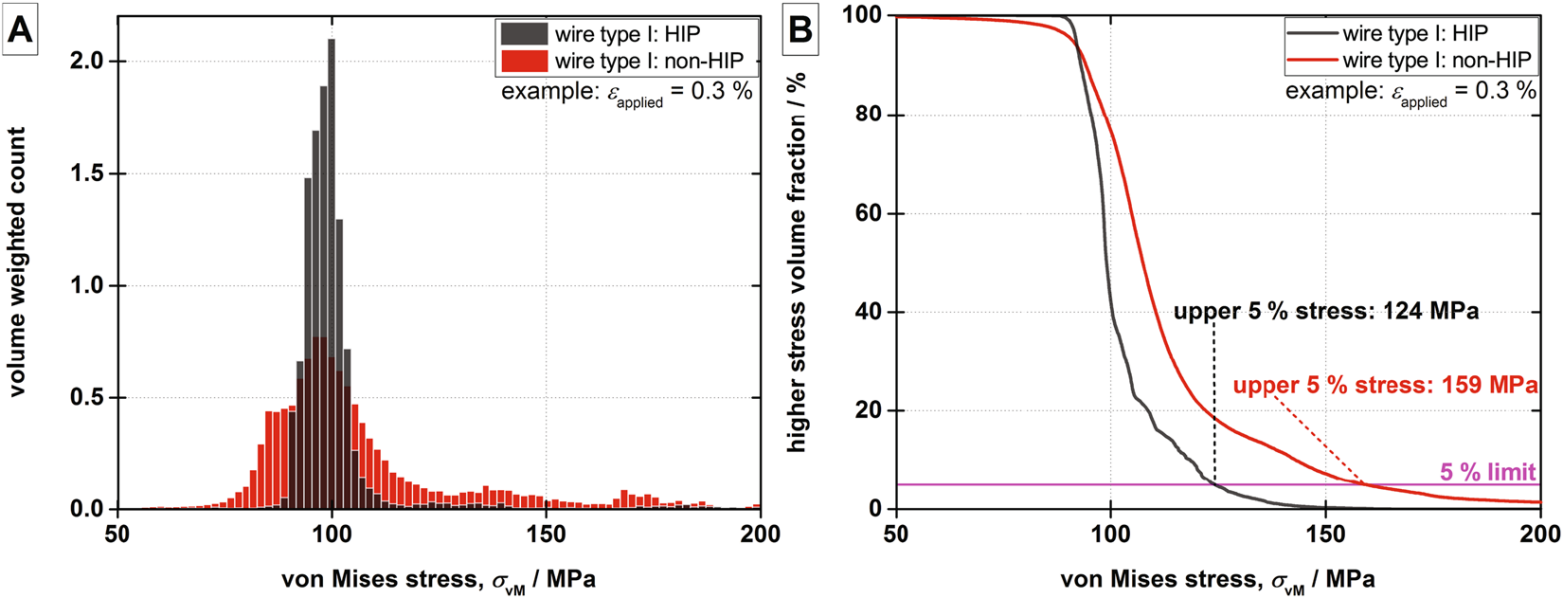
Post-processing of the FEM results. Exemplary histogram of the von Mises stress weighted on the element volume for wire type I at 0.3% applied strain (**A**) and corresponding cumulative von Mises stress distribution (**B**).

One side of the disk is fixed while a displacement is imposed as a boundary condition on the other to apply a mechanical strain. A physics controlled tetrahedral mesh with a minimal feature size of 0.05 μm and a maximal feature size of 10 μm is employed. The mesh sizes are verified through a mesh density study. For each mesh element in the Nb_3_Sn filament bundles, the element volume and the von Mises stress depending on the applied strain are assembled into a volume-weighted von Mises stress histogram. In general, comparing the HIP and non-HIP cases, the concentration of mechanical stress around the increased number of void ellipsoids leads to a broadening and shift towards higher von Mises stresses as shown exemplary in Fig. 6A for 0.3% applied strain in wire type I.

For given von Mises stresses, calculating the fraction of the volume that is at higher stress yields the volume-weighted cumulative von Mises stress map. The intersection of the this curve with the horizontal 5% line, gives the “upper 5% stress”, *σ*_upper5%_. Thus the *σ*_upper5%_ at the critical strain means that in 5% of the superconductor volume the stress locally exceeds the critical stress limits of the material reducing the total critical current of the wire by 5%. Assuming that 5% irreversible reduction of *I*_c_ is determined by damage in 5% of the filaments, this criterion is identical to the 5% critical current degradation limit used to determine the irreversible strains *ε*_irr_ in the electro-mechanical measurements. As mentioned in the Methods, “Electro-mechanical measurements” subsection, a threshold value of 5% has been proven suitable for modeling and furthermore it is in line with common limits employed in other types of electro-mechanical measurements. As an example of this method, wire type I, HIP and non-HIP are compared at 0.3% of applied strain in Fig. 6B. A broadening and shift towards higher stress of the cumulative von Mises stress map can be observed in the non-HIP treated case. The upper 5% stress is strongly increased from 124 MPa in the HIP case to 159 MPa in the non-HIP case, clearly highlighting the effect of the voids as stress concentrators.
